# Enzymes Are Enriched in Bacterial Essential Genes

**DOI:** 10.1371/journal.pone.0021683

**Published:** 2011-06-28

**Authors:** Feng Gao, Randy Ren Zhang

**Affiliations:** 1 Department of Physics, Tianjin University, Tianjin, China; 2 Center for Molecular Medicine and Genetics, School of Medicine, Wayne State University, Detroit, Michigan, United States of America; Laurentian University, Canada

## Abstract

Essential genes, those indispensable for the survival of an organism, play a key role in the emerging field, synthetic biology. Characterization of functions encoded by essential genes not only has important practical implications, such as in identifying antibiotic drug targets, but can also enhance our understanding of basic biology, such as functions needed to support cellular life. Enzymes are critical for almost all cellular activities. However, essential genes have not been systematically examined from the aspect of enzymes and the chemical reactions that they catalyze. Here, by comprehensively analyzing essential genes in 14 bacterial genomes in which large-scale gene essentiality screens have been performed, we found that enzymes are enriched in essential genes. Essential enzymes have overrepresented ligases (especially those forming carbon-oxygen bonds and carbon-nitrogen bonds), nucleotidyltransferases and phosphotransferases, while have underrepresented oxidoreductases. Furthermore, essential enzymes tend to associate with more gene ontology domains. These results, from the aspect of chemical reactions, provide further insights into the understanding of functions needed to support natural cellular life, as well as synthetic cells, and provide additional parameters that can be integrated into gene essentiality prediction algorithms.

## Introduction

Essential genes are those indispensable for the survival of an organism under certain conditions. Studies on essential genes play an important role in the emerging field, synthetic biology [1]. An important concept of synthetic biology is the chassis, which is the minimal genome capable of supporting a self-replicating organism. The minimal gene set, composed of all essential genes for an organism, is necessary to finally build a chassis in which interchangeable standardized gene circuits can be placed to create organisms with desirable traits [2], [3], [4], [5], [6]. Identification and characterization of essential genes have important practical implications, e.g., in identifying bacterial drug and vaccine targets; they can also enhance our understanding of basic biology, such as fundamental functions needed to support a cellular life.

There has been great advancement in gene essentiality studies in the past few years. For instance, 5 years after we created DEG, a database of essential genes [7], the number of experimentally determined bacterial essential genes has increased for more than tenfold [8]. This is mainly due to the increased ability of genome-wide gene essentiality screens in bacteria. So far, genome-wide or large-scale gene essentiality screens have been performed in 14 genomes [8]. Many studies have been performed to examine the characteristics of bacterial essential genes. For instance, it has been found that, compared to non-essential ones, bacterial essential genes tend to encode functions such as transcription, translation and replication [9], [10], tend to reside in the leading strand [11], are more evolutionarily conserved [12], and have different protein interaction network degrees [13].

Enzymes are the catalysts of biological systems, and most of them are proteins that catalyze specific chemical reactions. Enzymes have two striking characteristics, catalytic power and specificity. Enzymes can tremendously accelerate the rate of chemical reactions, and are highly selective for the substrates by only catalyzing very specific reactions. Therefore enzymes are critical for almost all cellular activities. Nevertheless, essential genes have not been examined systematically from the aspect of enzymes.

Because of the critical functions of enzymes, we hypothesized that bacterial essential genes are enriched with enzymes, and some chemical reactions are preferentially catalyzed by essential enzymes. To test this hypothesis, we examined enzyme proportions, and enzyme type distribution in essential and non-essential genes, using all the 14 genomes that have large-scale gene essentiality screens performed. We found that essential genes have higher proportion of enzymes, and that essential enzymes are enriched with ligases (especially those forming carbon-oxygen bonds and carbon-nitrogen bonds), nucleotidyltransferases and phosphotransferases, while have underrepresented oxidoreductases. These results provide further insights into the understanding of the functionalities of essential genes, and provide useful parameters that can be incorporated into gene essentiality prediction algorithms.

## Results and Discussion

### Enzymes are enriched in bacterial essential genes

**Figure 1:**
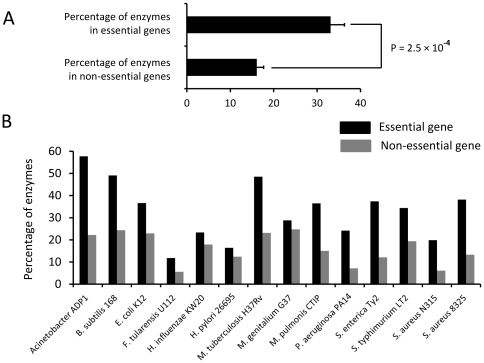
Enzymes are enriched in bacterial essential genes. (A) Averaged percentage of enzymes in essential and non-essential genes. (B) Percentages of enzymes in the 14 genomes in which large-scale gene essentiality screens have been performed.

Because of the critical functions of enzymes, we examined whether enzymes are enriched in bacterial essential genes. Based on the GenBank annotation, each enzyme has at least an Enzyme Commission number (EC code) [14], which specifies the chemical reactions that the enzyme catalyzes. So far, in 14 bacterial genomes, genome-wide or large-scale gene essentiality screens have been performed (Table 1). We then calculated the proportions of enzymes between essential and non-essential genes in these 14 genomes. On average, essential genes had more than 2-fold of enzymes than non-essential genes. The average percentages of enzymes in essential and non-essential genes were 33.07% and 16.15%, respectively (Fig. 1A). The Student's *t* test showed that the difference is statistically significant (p = 2.5×10^−4^). To confirm this, we also performed Mann-Whitney U test, which showed consistent result (p = 6.4×10^−4^). For all the 14 genomes, the enzyme proportions in essential genes were higher than those of non-essential genes (Fig. 1B). These results suggest that enzymes are enriched in bacterial essential genes.

**Table 1:** The data of essential genes used in the current study.

Organism	RefSeq	No. of total genes	No. of essential genes	References
*Acinetobacter* sp. ADP1	NC_005966	3307	499	[26]
*Bacillus subtilis* subsp. subtilis str. 168	NC_000964	4176	271	[10]
*Escherichia coli* K12	NC_000913	4144	700^a^	[27], [28]
*Francisella tularensis* subsp. novicida U112	NC_008601	1719	390	[35]
*Haemophilus influenzae* Rd KW20	NC_000907	1657	462	[32]
*Helicobacter pylori* 26695	NC_000915	1573	322^b^	[36]
*Mycobacterium tuberculosis* H37Rv	NC_000962	3988	614	[37]
*Mycoplasma genitalium* G37	NC_000908	475	378^c^	[38]
*Mycoplasma pulmonis* UAB CTIP	NC_002771	782	310	[39]
*Pseudomonas aeruginosa* UCBPP-PA14	NC_008463	5892	335	[40]
*Salmonella enterica* subsp. enterica serovar Typhi Ty2	NC_004631	4314	353	[41]
*Salmonella typhimurium* LT2	NC_003197	4423	230	[42]
*Staphylococcus aureus* subsp. aureus N315	NC_002745	2583	302	[29], [30], [31]
*Staphylococcus aureus* subsp. aureus NCTC 8325	NC_007795	2892	351	[43]

a Note that for the genome of NC_000913, ten essential genes without GI numbers and two essential genes with dead GI numbers (16129191 and 16130842) were excluded.

b Note that for the genome of NC_000915, one essential gene with dead GI number (15644641) was excluded.

c Note that for the genome of NC_000908, two essential genes without GI numbers and one essential gene with dead GI number (13277519) were excluded.

### Essential genes have underrepresented oxidoreductases and overrepresented ligases

The EC number is specific to a chemical reaction, but does not specify genes. All enzymes can be classified into 1 of the 6 classes.

EC 1: oxidoreductases, which catalyze oxidation and/or reduction reactions.
EC 2: transferases, which transfer a functional group, such as a methyl group.
EC 3: hydrolases, which catalyze the hydrolysis of various bonds.
EC 4: lyases, which cleave various bonds by means other than hydrolysis and oxidation.
EC 5: isomerases, which catalyze isomerization.
EC 6: ligases, which join two molecules with covalent bonds.

**Figure 2:**
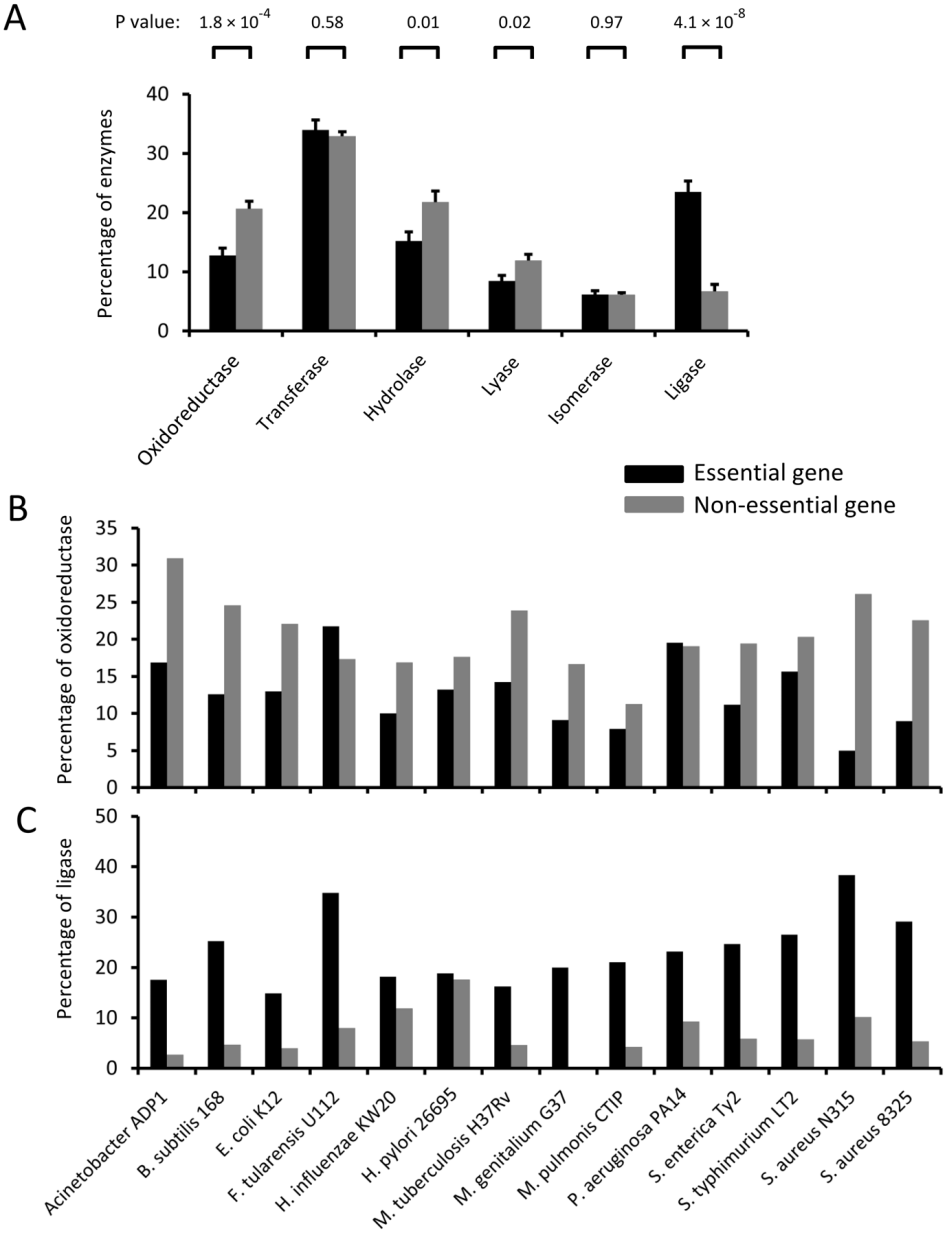
Distribution of enzyme classes for essential and non-essential genes. (A) Enzymes are classified into 1 of the 6 classes, oxidoreductases, transferases, hydrolases, lyases, isomerases and ligases. The proportion of oxidoreductases is significantly lower, while that of ligases is significantly higher in essential than in non-essential genes. (B) Percentages of oxidoreductases and (C) ligases in the 14 genomes studied.

We then examined the distribution of the 6 enzyme types among essential and non-essential genes. Ligases were highly enriched in essential genes (p = 4.1×10^−8^). On average, essential genes had more than 3 fold of ligases than non-essential genes. The percentages of ligases for essential and non-essential genes were 23.46% and 6.71%, respectively. Conversely, oxidoreductases were overrepresented in non-essential genes (p = 1.8×10^−4^). The percentages for oxidoreductases for essential and non-essential genes were 12.78% and 20.61%, respectively. The difference of proportions for other enzyme types, transferases, lyases and isomerases, was less significant (Fig. 2A). Essential genes tended to have lower proportion of hydrolases (p<0.05). Twelve of the 14 genomes had a higher proportion of oxidoreductases in non-essential genes (Fig. 2B), and in all the 14 genomes, the proportions of ligases were higher in essential genes (Fig. 2C). These results suggest that ligases are overrepresented and oxidoreductases are underrepresented in essential genes.

### Over and under-represented second level enzyme types in essential genes

Enzymes are broadly classified into 6 classes, and within each class, there are many subclasses. For instance, ligases are further classified into 6 subclasses, which include reactions forming carbon-oxygen bonds, carbon-sulfur bonds, carbon-nitrogen bonds, carbon-carbon bonds, phosphoric ester bonds and nitrogen-metal Bonds. We then examined the subclass distribution, and found that 4 subclasses were either over or under represented with statistical significance in essential genes.

**Figure 3:**
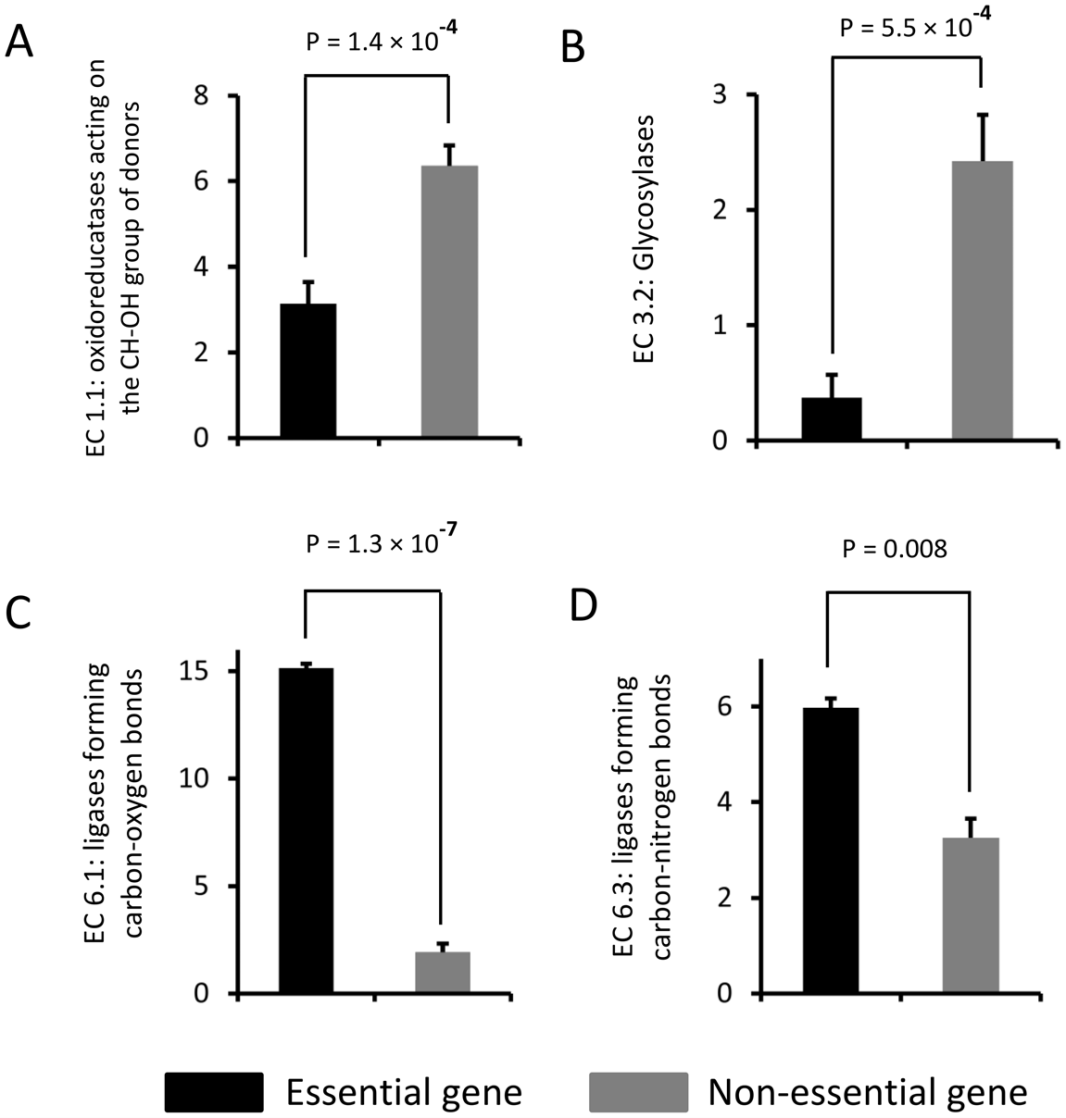
Significantly over- and under-represented second level enzyme types in essential genes. Significantly over- and under-represented second level enzyme types of (A) EC 1.1: oxidoreducatases acting on the CH-OH group of donors (B) EC 3.2: glycosylases (C) EC 6.1: ligases forming carbon-oxygen bonds and (D) EC 6.3: ligases forming carbon-nitrogen bonds, in essential genes.

Essential genes had underrepresented enzymes of the following types (Fig. 3). EC 1.1: oxidoreducatases acting on the CH-OH group of donors (ratio between essential and non-essential genes = 0.49) and EC 3.2: glycosylases (ratio = 0.15). Essential genes had overrepresented enzymes of the following types. EC 6.1: ligases forming carbon-oxygen bonds (ratio = 7.9) and EC 6.3: ligases forming carbon-nitrogen bonds (ratio = 1.8). Essential genes also tended to have higher proportion of transferases transferring phosphorus-containing groups (EC 2.7) (p = 0.01). The overrepresentation and underrepresentation of these enzyme types reveal important characteristics of essential genes from the aspect of chemical reactions.

### Enriched third level enzyme types in essential genes

EC codes have 4 levels, with progressively finer classification of enzyme types. For instance, EC 2.7.10.1 represents receptor protein-tyrosine kinase, which is a kind of protein-tyrosine kinases (EC 2.7.10), while the latter belongs to transferases (EC 2) that transferring phosphorus-containing groups (EC 2.7). Therefore it is necessary to examine further detailed enzyme classification for the second level enzymes that showed significant differences. However, with finer classification, the enzyme number becomes lower, and it is more stringent to reach significance with statistical tests. We found that following 3 enzyme types were especially enriched in essential genes.

**Figure 4:**
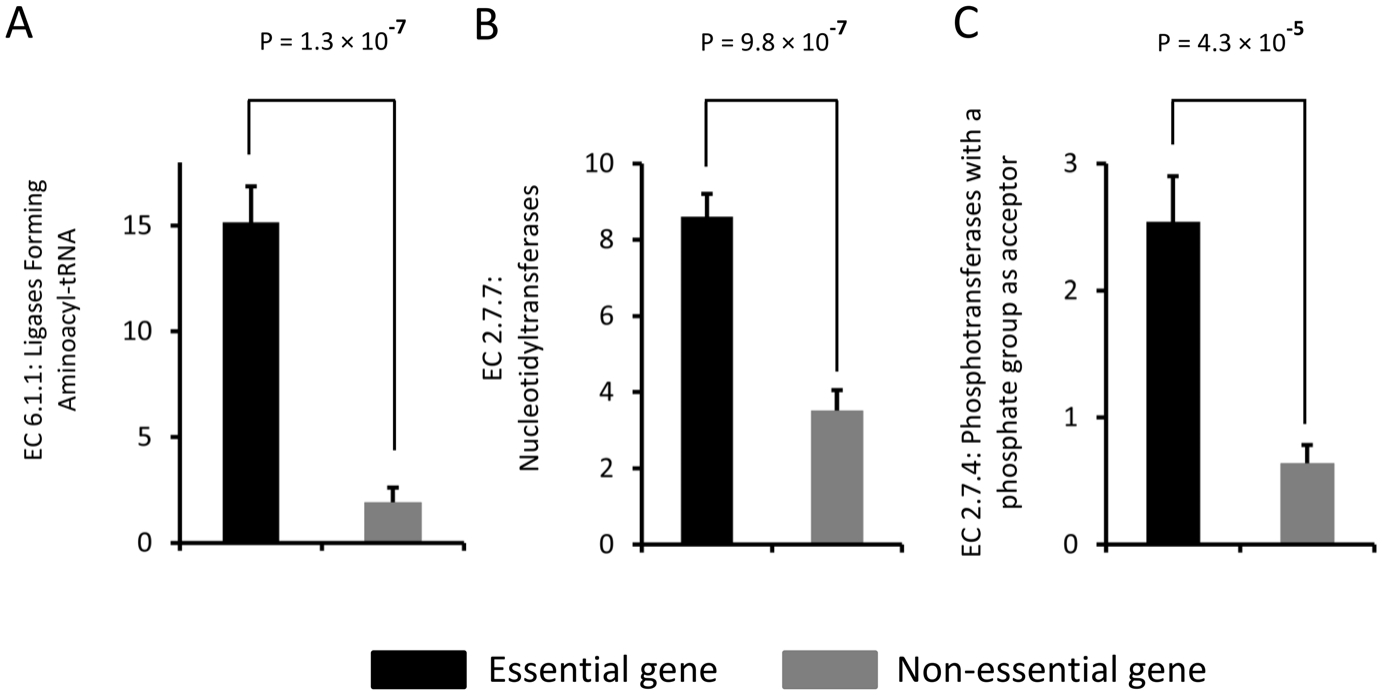
Significantly enriched third level enzyme types in essential genes. Significantly enriched third enzyme types of (A) EC 6.1.1: ligases forming aminoacyl-tRNA (B) EC 2.7.7: nucleotidyltransferases (C) EC 2.7.4: phosphotransferases with a phosphate group as acceptor, in essential genes.

Compared to non-essential genes, ligases forming aminoacyl-tRNA (EC 6.1.1) were enriched in essential genes. The proportion of this kind of ligases in essential genes was about 8 times of that in non-essential ones (p = 1.3×10^−7^). Similarly, in essential genes, the proportions of nucleotidyltransferases (EC 2.7.7) and phosphotransferases with a phosphate group as acceptor (EC 2.7.4) were both 2.5 times of those in non-essential genes, with highly statistical significance (Fig. 4).

### Essential enzymes are associated with more gene ontology domains

Gene ontology (GO) describes gene products with controlled vocabulary in a species-independent manner [15]. To describe functions, GO uses 3 domains (structured vocabularies), cellular components, molecular functions and biological processes. A cellular component is the part of a cell or its extracellular environment; the molecular function refers to the elemental activities of a gene product at the molecular level and the biological process refers to operations or sets of molecular events with a defined beginning and end, pertinent to the functioning of integrated cells [15].

**Figure 5:**
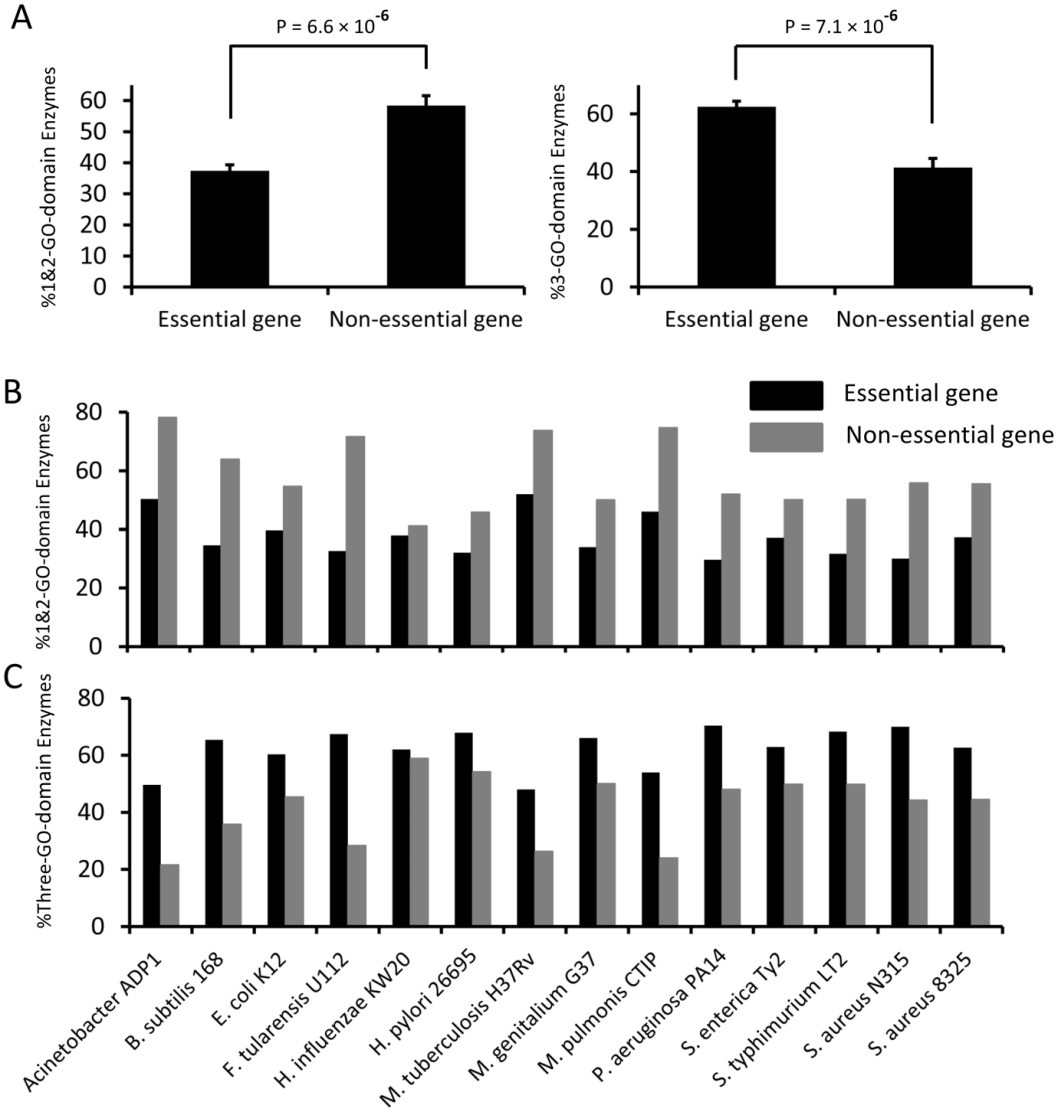
Essential enzymes are associated with more gene ontology domains. Based on gene ontology, genes can be assigned 3 GO domains, molecular function, biological process and cellular component, which are independent of each other. (A) Essential enzymes have higher proportion of 3-GO-domain and lower proportion of 1&2-GO-domain containing genes. (B) Percentages of 1&2-GO-domain and (C) 3-GO-domain containing genes in essential and non-essential enzymes.

A gene can be associated with 1 or more GO domains. We used Blast2GO [16] to assign GO terms to all genes, and calculated the proportion of genes with different GO domain numbers. We found that essential enzymes tended to associate with all the 3 GO domains. The percentages of 1 GO domain essential and non-essential enzymes were 1.96% and 7.05% (p = 0.09); those of 2 GO domain were 35.54% and 51.33% (p = 2.6×10^−5^). When combined, the percentages of either 1 or 2 GO domain essential and non-essential enzymes were 37.50% and 58.38%, respectively (p = 6.6×10^−6^) (Fig. 5A). In contrast, essential enzymes had higher proportion of genes associated with all 3-GO domains (p = 7.1×10^−6^). The percentages of 3 GO domain essential and non-essential enzymes were 62.50% and 41.48%, respectively (Fig. 5A). The observations that compared to non-essential ones, essential enzymes had lower proportion of genes associated with either 1 or 2-GO domains, but higher proportion of 3-GO domains, hold for all the genomes studied (Fig. 5B and C). The observation that essential enzymes tend to be associated with more gene ontology domains seems to reflect the multi-functional features of essential enzymes, consistent with their lethality phenotypes.

Every organism and the complex cellular activities that support its survival is an end product of evolution of millions of years. Life, to a large degree, can be regarded as a series of chemical reactions. Then enzymes that catalyze these chemical reactions must play a special role in the survival of an organism. By definition, essential genes are those absolutely needed for the survival of an organism. Of note, experimentally determined essential genes rely on specific conditions, such as minimal medium [17]. In this study, we calculated the enzyme proportions in essential genes comprehensively in 14 bacterial genomes that have large-scale gene essentiality screens performed. Indeed, essential genes had a higher proportion of enzymes.

A caveat in interpreting the results is the annotation bias, e.g., in the GenBank. To minimize the annotation bias, we analyzed the dataset by only keeping well-characterized genes and removing genes with the annotations of ‘putative’, ‘probable’, ‘possible’, ‘uncharacterized’, ‘conserved’, ‘hypothetical’, ‘unknown’ and ‘predicted’ functions. In 13 of the 14 genomes, essential genes had higher enzyme proportions than non-essential genes. On average, enzyme percentages were 41.27% and 29.52% for essential and non-essential genes, respectively (P = 0.019). Therefore, enzymes were still enriched in essential genes after largely correcting the annotation biases, which did seem to exist.

**Figure 6:**
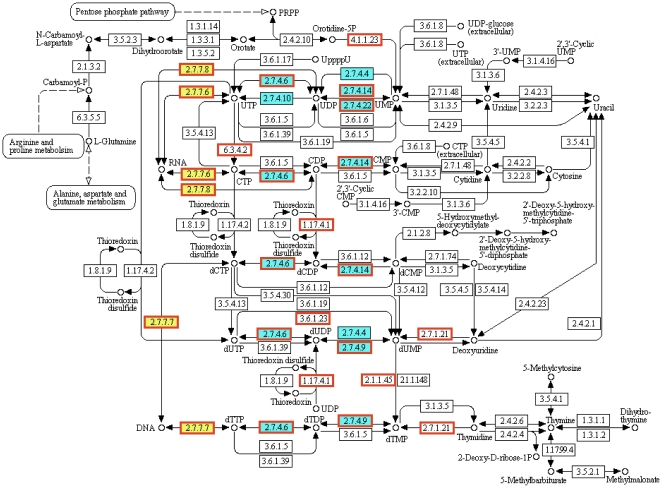
Overrepresentation of EC 2.7.7 and EC 2.7.4 in essential genes involved in pyrimidine metabolism pathway. Part of the pyrimidine metabolism map is shown. The DAVID database was used to identify pathways in which essential genes are enriched, using *E. coli* as an example. Red rectangles denote reactions involving essential genes; filled yellow and blue boxes represent EC 2.7.7 (nucleotidyltransferase) and EC 2.7.4 (phosphotransferase), respectively.

Enzymes encoded by essential genes are essential enzymes. Therefore, enzymes can be classified as either essential enzymes or non-essential enzymes. The two types of enzymes have distinct distributions in terms of chemical reactions that they catalyze. Essential enzymes are enriched with ligases (EC 6), while non-essential enzymes are enriched with oxidoreductases (EC 1). There are many kinds of ligases, and the essential ligases were mainly the ones forming carbon-oxygen bonds (EC 6.1) and carbon-nitrogen bonds (EC 6.3). The main ligases responsible for the enrichment of the former are those forming aminoacyl-tRNA (EC 6.1.1), most of which are various tRNA synthetases; the ones mainly responsible for the enrichment of the latter (EC 6.3) are acid-amino-acid ligases (EC 6.3.2), which are involved in peptide synthases. Essential genes had higher proportion of transferases transferring phosphorus-containing groups (EC 2.7), and this was mainly due to the enrichment of nucleotidyltransferases (EC 2.7.7) and phosphotransferases (EC 2.7.4). The enriched nucleotidyltransferases are mainly DNA-directed RNA polymerase (EC 2.7.7.6) and DNA-directed DNA polymerase (EC 2.7.7.7). The enriched phosphotransferases are mainly cytidylate kinases (EC 2.7.4.14), guanylate kinases (EC 2.7.4.8) and dTMP kinases (EC 2.7.4.9), which are involved in metabolisms of purine and pyrimidine. Indeed, by using the DAVID database [18], we found that in pathways of aminoacyl-tRNA biosynthesis, purine and pyrimidine metabolism, essential genes were especially enriched. In *E. coli*, for example, most essential genes in the pyrimidine metabolism pathway belong to EC 2.7.7 and EC 2.7.4 (Fig. 6).

One important feature of the EC code is that it is specific to chemical reactions, but independent of species and genes. In other words, different genes from different species can have the same EC code. Therefore, it is an ideal platform to compare chemical reactions between essential and non-essential enzymes across species. Because it is labor-intensive and time-consuming to identify essential genes experimentally, in the past years, much effort has been devoted to developing algorithms for gene essentiality prediction [9], [13], [19], [20], [21], [22], [23], [24], [25]. Gene features that have been used include, for instance, expression levels [19], evolutionary rates [20], connectivity in protein interaction networks [25] and codon usage [13]. Nevertheless, enzyme annotation, to the best of our knowledge, has not been used for predicting essential genes. Therefore, the results that essential genes are enriched with enzymes and that certain chemical reactions are either over or under-represented in essential enzymes will provide additional parameters for *in silico* identification of essential genes.

In summary, by comprehensively analyzing essential genes in bacterial genomes in which large-scale gene essentiality screens have been performed, we found that enzymes are enriched in essential genes. Essential enzymes have overrepresented ligases (especially those forming carbon-oxygen bonds and carbon-nitrogen bonds), nucleotidyltransferases and phosphotransferases, while have underrepresented oxidoreductases. Furthermore, essential enzymes tend to associate with more GO domains. These results, from the aspect of chemical reactions, provide further insights into the understanding of functions needed to support natural cellular life, as well as synthetic cells, and provide additional parameters that can be integrated into gene essentiality prediction algorithms.

## Materials and Methods

Essential-gene records in DEG 6.0 were used in the current study. Fourteen genomes in which genome-wide or large-scale gene essentiality screens that have been performed were used (Table 1). Among the 14 genomes, in *Acinetobacter* sp. ADP1 [26] and *B. subtilis* str. 168 [10], essential genes were obtained by targeted gene deletions. In *E. coli* K12, two genome-wide gene essentiality studies were performed. One study generated comprehensively single gene knockout mutants [27], and other study obtained essential genes by transposon mutagenesis [28]. The results from both studies were pooled in the current analysis. For the genome of *S. aureus* subsp. N315, results from 3 large scale studies [29], [30], [31] were pooled. For the genome of *H. influenzae* Rd KW20 [32], we only included genes with no transposon insertions as essential genes, to be consistent with the definition of other studies. In other genomes, *F. tularensis* subsp. U112, *H. pylori* 26695, *M. tuberculosis* H37Rv, *M. genitalium* G37, *M. pulmonis* UAB CTIP, *P. aeruginosa* PA14, *S. enterica* Typhi Ty2, *S. typhimurium* LT2 and *S. aureus* NCTC 8325, gene essentiality screens were performed by transposon mutagenesis.

The GenBank annotation files were downloaded from the NCBI FTP server (ftp://ftp.ncbi.nih.gov/genomes/Bacteria) on August 21, 2010. The EC code annotation available in GBK files were used to assign enzyme types. Student *t* tests were performed to compare the average proportions of enzymes or enzyme classes between essential and non-essential genes, unless indicated otherwise. Blast2GO [16] was used to assigned GO terms to all genes. Annotations for *E. coli* by Blast2GO and EcoCyc [33] were found to be consistent. The database for annotation, visualization and integrated discovery (DAVID) [18] was used to identify pathways in which essential genes are enriched. The pyrimidine metabolism pathway map (Fig. 6) was modified from the one available at KEGG [34]. Values are presented as mean ± s.e.m. P values less than 0.05 were considered statistically significant.
